# *Candida albicans* Gene Deletion with a Transient CRISPR-Cas9 System

**DOI:** 10.1128/mSphere.00130-16

**Published:** 2016-06-15

**Authors:** Kyunghun Min, Yuichi Ichikawa, Carol A. Woolford, Aaron P. Mitchell

**Affiliations:** Department of Biological Sciences, Carnegie Mellon University, Pittsburgh, Pennsylvania, USA; University of Michigan

**Keywords:** *Candida albicans*, genetics

## Abstract

Clustered regularly interspaced short palindromic repeat (CRISPR) and CRISPR-associated gene 9 (CRISPR-Cas9) systems are used for a wide array of genome-editing applications in organisms ranging from fungi to plants and animals. Recently, a CRISPR-Cas9 system has been developed for the diploid fungal pathogen *Candida albicans*; the system accelerates genetic manipulation dramatically [V. K. Vyas, M. I. Barrasa, and G. R. Fink, Sci Adv 1(3):e1500248, 2015, http://dx.doi.org/10.1126/sciadv.1500248]. We show here that the CRISPR-Cas9 genetic elements can function transiently, without stable integration into the genome, to enable the introduction of a gene deletion construct. We describe a transient CRISPR-Cas9 system for efficient gene deletion in *C. albicans*. Our observations suggest that there are two mechanisms that lead to homozygous deletions: (i) independent recombination of transforming DNA into each allele and (ii) recombination of transforming DNA into one allele, followed by gene conversion of the second allele. Our approach will streamline gene function analysis in *C. albicans*, and our results indicate that DNA can function transiently after transformation of this organism.

**IMPORTANCE** The fungus *Candida albicans* is a major pathogen. Genetic analysis of this organism has revealed determinants of pathogenicity, drug resistance, and other unique biological features, as well as the identities of prospective drug targets. The creation of targeted mutations has been greatly accelerated recently through the implementation of CRISPR genome-editing technology by Vyas et al. [Sci Adv 1(3):e1500248, 2015, http://dx.doi.org/10.1126/sciadv.1500248]. In this study, we find that CRISPR elements can be expressed from genes that are present only transiently, and we develop a transient CRISPR system that further accelerates *C. albicans* genetic manipulation.

## Abstract (precis)

The fungus *Candida albicans* is a major pathogen. Genetic analysis of this organism has revealed determinants of pathogenicity, drug resistance, and other unique biological features, as well as the identities of prospective drug targets. The creation of targeted mutations has been greatly accelerated recently through the implementation of CRISPR genome-editing technology by Vyas et al. [Sci Adv 1(3):e1500248, 2015, http://dx.doi.org/10.1126/sciadv.1500248]. In this study, we find that CRISPR elements can be expressed from genes that are present only transiently, and we develop a transient CRISPR system that further accelerates *C. albicans* genetic manipulation.

## INTRODUCTION

*Candida albicans* is a human commensal that lives on mucosal surfaces. It is thus poised to proliferate when conditions are permissive, which can lead to mucosal and invasive infections (1, 2). Molecular genetics of *C. albicans* can facilitate the discovery of antifungal agents and elucidation of pathogenesis mechanisms. However, the creation of homozygous deletion mutants in this diploid organism remains a slow step in gene function analysis.

Recently, Vyas and colleagues greatly accelerated *C. albicans* genetic analysis by adapting a clustered regularly interspaced short palindromic repeat (CRISPR) and CRISPR-associated gene 9 (CRISPR-Cas9) system to rapidly create *C. albicans* homozygous mutants (3). CRISPR-Cas9 systems are adaptive immune systems in bacteria (4). The RNA-guided endonuclease activity of Cas9 has been harnessed for an array of genome-editing applications in organisms ranging from fungi to plants and animals (5, 6). The Vyas system consists of a *Candida* codon-optimized Cas9 nuclease gene (*CaCAS9*) and a single guide RNA (sgRNA) gene whose product directs Cas9 to cleave a specific site in the genome. The 20-base guide sequence from the sgRNA hybridizes to a genomic target, enabling CaCas9 to produce a double-strand break when the genomic target is followed by the protospacer-adjacent motif sequence, NGG. A CRISPR-induced double-strand break triggers homology-directed repair with mutagenic donor DNA so the genomic target can be precisely edited.

A potential rate-limiting step of the Vyas CRISPR system is the reliance upon genomic integration of the vector encoding CaCas9 and the sgRNA. In essence, the desired genome editing frequency may have been limited to the integration frequency of the CaCas9-sgRNA expression construct. We report here our finding that the CaCas9-sgRNA expression construct does not require genomic integration for functional activity. This observation led us to hypothesize that transient introduction of *CaCAS9* and the sgRNA gene might simplify *C. albicans* genome editing. We report here the evidence in support of this hypothesis and describe our modifications of the Vyas constructs that enable a transient introduction approach.

## RESULTS

### *Candida* CRISPR system function without stable integration.

**FIG 1:**
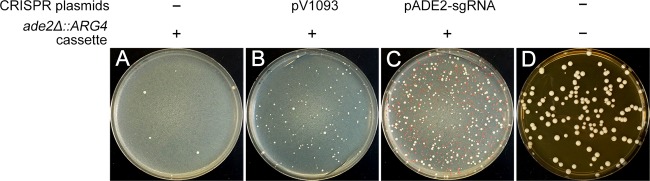
Production of *ade2Δ*/*ade2Δ* mutants by the *Candida* CRISPR system. An *ADE2* gene deletion template containing the *ARG4* cassette was transformed into BWP17 cells alone (A), along with the *CaCAS9* vector pV1093 (B), or along with the *CaCAS9*-and-sgRNA vector pADE2-sgRNA (C) and plated on medium lacking arginine to select for Arg^+^ transformants. (D) A 1/10,000 dilution of the negative control (no transforming DNA control) was plated on nonselective YPD plates.

We set out to use the CRISPR system for deletion and replacement of gene-sized DNA regions in *C. albicans*. The *ADE2* gene was targeted because homozygous *ade2* mutations result in a visible red phenotype, thus simplifying their identification. We used the CRISPR system vector pADE2-sgRNA, which has a functional *CaCAS9* and an sgRNA gene directed against *ADE2* (3). It also contains a *NAT* selection marker and 2-kb arms to enable homologous integration at the *ENO1* locus. Using Arg^−^ BWP17 cells, an *ADE2* gene deletion construct containing an *ARG4* cassette (the *ade2Δ*::*ARG4* template) was transformed with or without linearized pADE2-sgRNA DNA. We selected for Arg^+^ transformants, which selects for the *ade2Δ*::*ARG4* deletion rather than the CRISPR system vector, in order to assay the effects of the CRISPR system on integration. Transformation of the *ade2Δ*::*ARG4* template together with linearized pADE2-sgRNA DNA produced red Arg^+^ colonies at a high frequency, whereas transformation of the *ade2Δ*::*ARG4* template alone produced only white Arg^+^ colonies (Fig. 1). These results suggested that the *Candida* CRISPR system induced the homozygous deletion of the *ADE2* open reading frame (ORF) (1.7 kb) and integration of the *ARG4* cassette. We then tested for integration of the pADE2-sgRNA plasmid segment by testing colonies for the *NAT* marker. Among thirty red and thirty white Arg^+^ colonies tested, none were nourseothricin resistant (Nat^R^ phenotype), indicating that they did not express the *NAT* marker. This result suggested that the pADE2-sgRNA DNA had not integrated into the genome. However, the abundance of red Arg^+^ transformants from the cotransformation samples argued that functional expression of *CaCAS9* and the sgRNA gene had occurred upon transformation.

### Transient CRISPR system for *C. albicans.*

**FIG 2:**
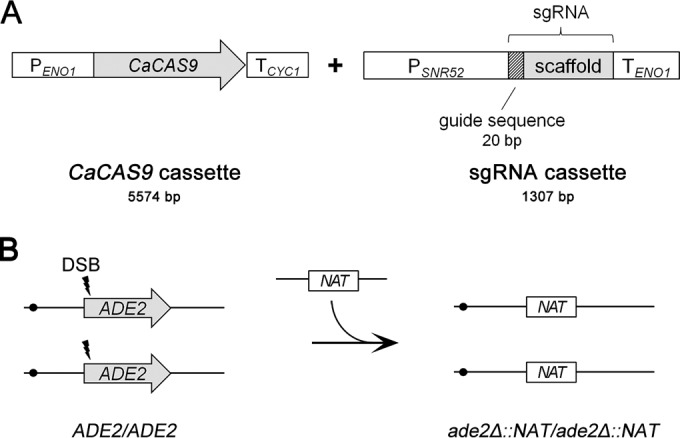
Transient CRISPR-Cas9 system. (A) Structures of *CaCAS9* and sgRNA expression cassettes. The *CaCAS9* gene was the Vyas codon-optimized version, expressed from the *ENO1* promoter and flanked by a *CYC1* terminator (3). The sgRNA was expressed under the *SNR52* promoter and contained the *ENO1* terminator (3). The guide sequence is 20 bp long and designed for each target sequence. (B) Schematic diagram of *ADE2* deletion strategy. The CRISPR system produces double-strand breaks (DSB) at target sequences. The double-strand breaks can be resolved by homology-directed repair with the gene deletion templates, which have 80-bp arms homologous to the target gene, to create a homozygous deletion of *ADE2*.

**FIG 3:**
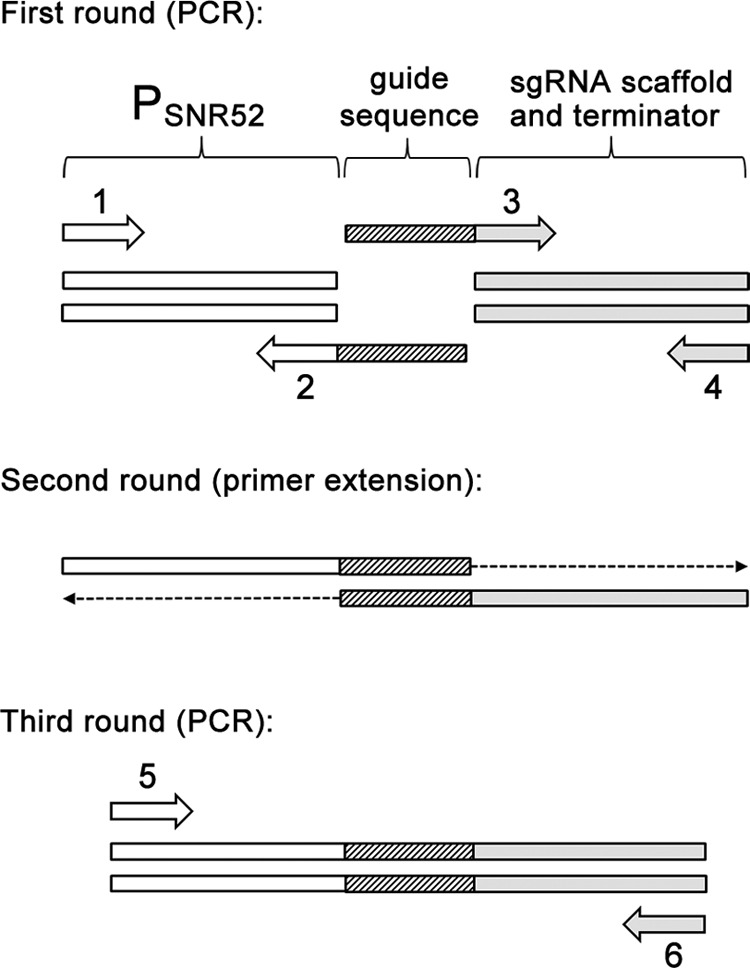
Construction of sgRNA expression cassette. Three DNA synthesis steps fuse DNA fragments comprising the *SNR52* promoter and the sgRNA scaffold. Chimeric primers 2 and 3 carry 20 complementary bases of guide sequence. In the first step, PCR is used to create two segments of the sgRNA gene. The *SNR52* promoter is amplified with primers 1 and 2; the sgRNA scaffold is amplified with primers 3 and 4. In the second step, primer extension is used to fuse the two PCR products, with chimeric extensions acting as primers. In the third step, PCR with nested primers 5 and 6 is used to amplify the final sgRNA expression cassette.

**FIG 4:**
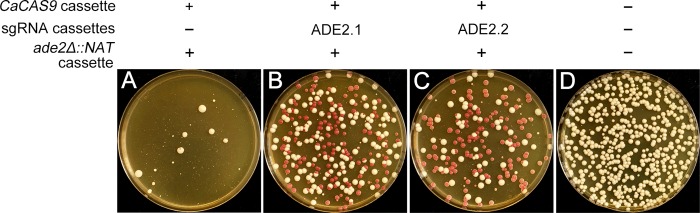
Functional assay for the *Candida* transient CRISPR system via recovery of *ade2Δ*/*ade2Δ* mutants. Strain SC5314 was transformed with the *ADE2* deletion template and *CaCAS9* expression cassette without an sgRNA cassette (A), with the ADE2.1 sgRNA cassette (B), or with the ADE2.2 sgRNA cassette (C) and plated on YPD+NAT. (D) A 1/10,000 dilution of the negative control (no transforming DNA control) was plated on nonselective YPD plates.

**FIG 5:**
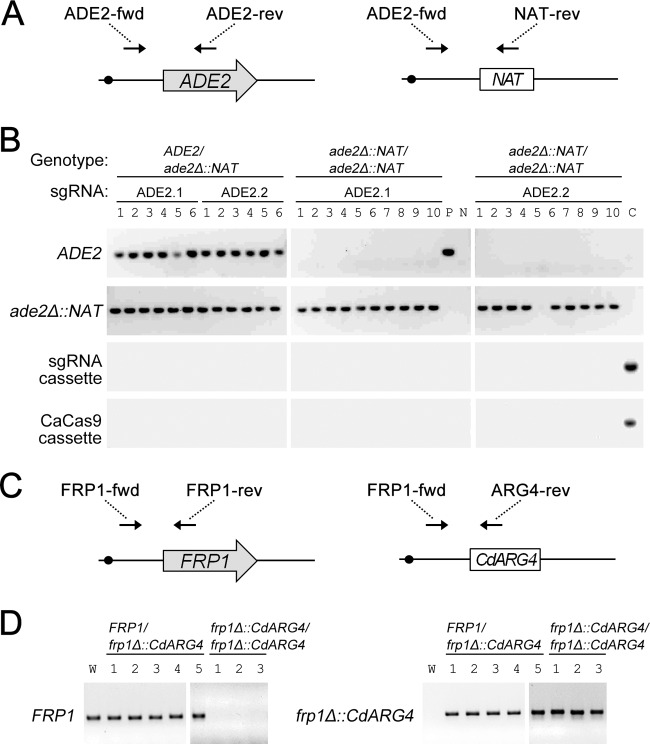
Genotype analysis of transient CRISPR system transformants. (A) PCR detection strategy for *ADE2* (left) and *ade2Δ*::*NAT* (right) alleles. The 5′-end-flanking primer ADE2-fwd was used with *ADE2* internal primer ADE2-rev to detect the *ADE2* allele and with *NAT* internal primer NAT-rev to detect the *ade2Δ*::*NAT* allele. (B) Allelic content of transformants. Genomic DNA was used for PCR genotyping from 12 white transformants, 20 red transformants, the parental strain (lane P), *CaCAS9*-and-sgRNA plasmid pV1093 (lane C), or a no-template control (lane N). Transformants with the ADE2.1 or ADE2.2 sgRNA gene were obtained, as indicated across the top. Genotyping reactions were for *ADE2*, *ade2Δ*::*NAT*, the sgRNA cassette, or an internal segment of *CaCAS9*, as indicated to the left. (C) PCR detection strategy for *FRP1* (left) and *frp1Δ*::*ARG4* (right) alleles. The 5′-end-flanking primer FRP1-fwd was used with *FRP1* internal primer FRP1-rev to detect the *FRP1* allele and with *CdARG4* internal primer ARG4-rev to detect the *frp1Δ*::*CdARG4* allele. (D) Allelic contents of transformants. Genomic DNA from 8 transformants, as well as wild-type strain SC5314 (lane W), was used for PCR genotyping. Genotyping reactions were for *FRP1* or *frp1Δ*::*CdARG4*, as indicated to the left of each gel image.

In view of the results described above, we tested a *C. albicans* transient CRISPR system that targets the *ADE2* gene. This system consisted of separate *CaCAS9* and sgRNA expression cassettes (Fig. 2A). The *CaCAS9* expression cassette was PCR amplified from the plasmid pV1093 (3). The sgRNA expression cassette was constructed through single-joint PCR (Fig. 3). We used the sgRNA ADE2.1 guide RNA sequence (as designed and used by Vyas et al. [3]), which directs CaCas9 activity to a site 41 bp downstream from the *ADE2* start codon. An *ade2Δ*::*NAT* construct served as a template to create a deletion of the *ADE2* target gene (Fig. 2B). The *ade2Δ*::*NAT* construct had 80-bp arms homologous to sequences upstream or downstream from the *ADE2* coding region, and the site targeted by the sgRNA ADE2.1 guide RNA was immediately adjacent to one *ade2Δ*::*NAT* deletion endpoint and 1.7 kbp from the other. The *ade2Δ*::*NAT* construct was cotransformed into strain SC5314 with PCR products for *CaCAS9* and the sgRNA ADE2.1 gene, and Nat^R^ transformants were selected. Transformants were recovered at a frequency of 4.7 × 10^−5^ (Table 1). This version of the CRISPR system produced red transformants at high frequency (Fig. 4; Table 1), and PCR genotyping of 10 such colonies verified that they carried only *ade2Δ*::*NAT* alleles (Fig. 5A and B). The production of red colonies among the transformants required both *CaCAS9* and sgRNA (Table 1). In the absence of either CRISPR component, the frequency of Nat^R^ transformants was reduced more than 10-fold and only white transformants were recovered (Table 1). We used PCR to determine whether *CaCAS9* or the sgRNA expression cassette was detectable in genomic DNA of the transformants. Among 10 transformants, we detected no signal from *CaCAS9* with internal primers and no signal from the sgRNA expression cassette (Fig. 5B). These results indicated that intact CRISPR component cassettes had not integrated into the genome. The effect of *CaCAS9* and the sgRNA expression cassette on the transformation outcome argued that the CRISPR system had functioned at a critical time during transformation. Overall, our results indicate that a transient presence of intact CRISPR component genes is sufficient to promote recombination events that result in gene deletion.

**TABLE 1:** Transformation frequencies with the transient CRISPR system^a^

*CaCAS9* expression cassette	sgRNA expression cassette	*ade2*Δ::*NAT* deletion construct	NAT resistance frequency	Homozygous *ADE2* deletion frequency
−	−	+	1.4 × 10^−6^	0.0
+	−	+	8.3 × 10^−7^	0.0
−	*ADE2.1*	+	8.3 × 10^−7^	0.0
+	*ADE2.1*	+	4.7 × 10^−5^	2.1 × 10^−5^
+	*ADE2.1*	−	0.0	0.0
−	*ADE2.2*	+	1.3 × 10^−6^	0.0
+	*ADE2.2*	+	2.1 × 10^−5^	1.4 × 10^−5^
+	*ADE2.2*	−	0.0	0.0
−	−	−	0.0	0.0

a Transformation frequency was calculated as the ratio of the number of colonies on YPD+NAT medium divided by the number on nonselective YPD medium. Each transformation experiment was repeated four times.

We sought to determine whether the guide RNA target site could be distant from both deletion endpoints. To that end, we tested the sgRNA ADE2.2 genomic target site, which was located 912 bp downstream from the start codon, a site that is 0.9 kbp from each *ade2Δ*::*NAT* deletion endpoint. We observed that *ade2Δ*::*NAT* deletion events were stimulated to a comparable extent by sgRNA ADE2.2 and ADE2.1 guide RNA genes (Table 1; Fig. 4). PCR genotyping of red transformants made with the ADE2.2 sgRNA gene verified that 9 of 10 carried only *ade2Δ*::*NAT* alleles and failed to detect the presence of *CaCAS9* or the sgRNA expression cassette (Fig. 5A and B). These results indicate that recombination sites can lie far from the site of CRISPR-directed double-strand break formation in *C. albicans*, as has been found previously in yeast and mouse (7, 8).

In order to determine whether the transient CRISPR system would allow homozygous deletion recovery for loci besides *ADE2*, we used the system to delete the *FRP1* gene in the SN152 strain background (9), using *frp1Δ*::*CdARG4* (with *ARG4* from *Candida dubliniensis*) as the template sequence. We used a *yhb5Δ*/*Δ* strain (10) as a transformation recipient because we hypothesized that Yhb5 and Frp1 may have related functions. Targeting was accomplished with an sgRNA sequence developed by Vyas et al. (3) that targets the 5′ end of the *FRP1* coding region, directing cleavage adjacent to one template recombination site and 1.6 kbp from the other. Among 8 Arg^+^ transformants, three yielded only an *frp1Δ*::*CdARG4* PCR product, as expected for homozygous deletion mutants (Fig. 5C and D). These results indicate that homozygous deletion mutants can be recovered with the transient CRISPR system at two loci, *FRP1* and *ADE2*, and can be employed with the popular SN152 strain background.

### Independent integration and allelic gene conversion.

**FIG 6:**
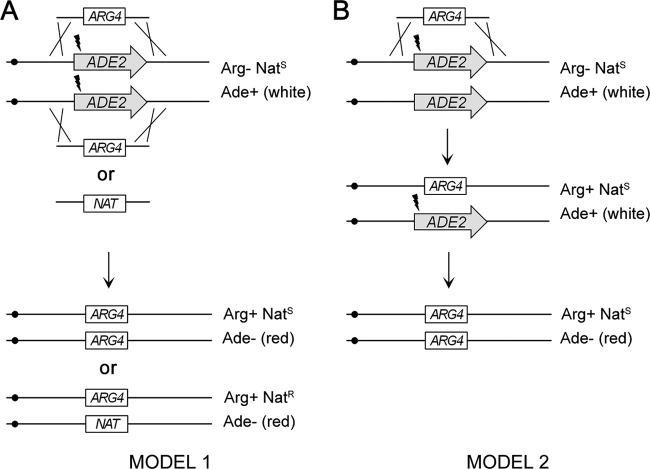
Two models for the CRISPR-enabled generation of a homozygous *ADE2* deletion. We depict an experiment in which mixed *ade2Δ*::*NAT* and *ade2Δ*::*ARG4* deletion templates are transformed along with the *CaCAS9* and sgRNA ADE2.1 PCR products. Double-strand breaks are represented by lightning bolts. (A) In the independent integration model, homozygotes arise from independent recombination events that integrate two repair template molecules, one per allele. Assuming that *ade2Δ*::*NAT* and *ade2Δ*::*ARG4* deletion templates have equal chances of integration into *ADE2* alleles (which we designate *ADE2A* and *ADE2B*), then we predict equal numbers of red homozygous transformants of genotypes *ade2AΔ*::*ARG4*/*ade2BΔ*::*ARG4*, *ade2AΔ*::*ARG4*/*ade2BΔ*::*NAT*, *ade2AΔ*::*NAT*/*ade2BΔ*::*ARG4*, and *ade2AΔ*::*NAT*/*ade2BΔ*::*NAT.* Only the first three classes will be detected among Arg^+^ transformants, so the ratio of red Arg^+^ Nat^R^ transformants to red Arg^+^ Nat^S^ transformants should be 2:1. (B) In the allelic gene conversion model, homozygotes arise from a recombination event to integrate a repair template into one allele, followed by a gene conversion or crossover event that copies the mutant allele sequence into the wild-type allele. This model predicts that none of the Arg^+^ red colonies will express the *NAT* marker, so the ratio of red Arg^+^ Nat^R^ transformants to red Arg^+^ Nat^S^ transformants should be 0:1.

We considered two models for the CRISPR-enabled generation of homozygous mutants. One model is that homozygotes arise from independent recombination events that integrate two repair template molecules, one per allele (Fig. 6A). A second model is that homozygotes arise from a recombination event that integrates a repair template into one allele, followed by a gene conversion or crossover event that transfers the mutant allele sequence into the remaining wild-type allele (Fig. 6B). These models are not mutually exclusive; it is possible that both mechanisms occur. We used transformation of two differentially marked repair templates into strain BWP17 to test these models. We mixed *ade2Δ*::*NAT* and *ade2Δ*::*ARG4* deletion cassettes in a 1:1 molar ratio and transformed BWP17 cells with the mixture, along with the *CaCAS9* and sgRNA ADE2.1 PCR products. Arg^+^ transformants were selected, and red colonies were scored for the *NAT* marker. The first model predicts that two-thirds of Arg^+^ red colonies will express the *NAT* marker and be Nat^R^; the second model predicts that none of the Arg^+^ red colonies will be Nat^R^. In three independent experiments, we routinely recovered Arg^+^ Nat^R^ transformants (Table 2), thus indicating that independent integration (model 1) does occur. However, fewer than two-thirds of the Arg^+^ transformants were Nat^R^, thus arguing that allelic recombination (model 2) occurs as well. The biased recovery of transformants might reflect differing recombination potentials of the *ade2Δ*::*ARG4* and *ade2Δ*::*NAT* templates. However, that explanation was ruled out by a reciprocal selection in experiment 3: when Nat^R^ transformants were selected from a template cotransformation, we found that only a minority were Arg^+^ (Table 2). Hence, the transformants recovered were skewed in favor of the initially selected marker, regardless of whether it was *ARG4* or *NAT*. The skewed ratio of selected to unselected markers is significantly different from the 1:2 ratio that is expected from model 1, according to a chi-square test (*P* value = 0.0001). These results indicate that homozygous mutations sometimes arise from two independent integration events, one at each allele (model 1), and sometimes arise from a single integration event at one allele, followed by gene conversion or crossing over to alter the second allele (model 2).

**TABLE 2:** Recovery of unselected markers among Ade^−^ transformants^a^

Experiment	Transformant selection	No. of red transformants by phenotype^b^				Total no. of transformants
		Nat^S^	Nat^R^	Arg^−^	Arg^+^	
1	Arg^+^	17	3	NA	20	20
2	Arg^+^	15	8	NA	23	23
3	Arg^+^	26	15	NA	41	41
3	Nat^R^	NA	20	15	5	20

a *ade2*Δ::*ARG4* and *ade2*Δ::*NAT* deletion templates were mixed in a 1:1 molar ratio. BWP17 cells were transformed with the mixture along with the *CaCAS9* and sgRNA ADE2.1 PCR products. Transformants were selected through either an Arg^+^ or Nat^R^ phenotype, as indicated in the table. Red transformants were purified and scored for the unselected marker. The results of three independent experiments are shown.

b NA, not applicable because the selection did not allow recovery of the phenotype.

## DISCUSSION

This study presents three conclusions that will be useful for future application and interpretation of CRISPR-promoted mutants of *C. albicans*. First, we have found that the CRISPR components—the *CaCAS9* and sgRNA gene cassettes—can function when introduced transiently and without direct selection. This finding may streamline *Candida* CRISPR usage. The finding also implies that other genes might be introduced into *C. albicans* transiently for a variety of applications. Second, we have found that CRISPR-targeted cleavage can occur far from sites of recombination in *C. albicans*, as has been documented in other organisms. Hence, in *C. albicans*, internal cleavage in a coding region can be used to create a complete gene deletion. Third, we found that CRISPR-generated homozygous mutants can arise through gene conversion events between alleles, an outcome that emphasizes the need for mutant phenotype validation with this system. We discuss each point in turn, and then summarize with a brief overview of *C. albicans* transient CRISPR system usage.

The fact that CRISPR components can be functionally expressed transiently has several useful implications. Because genomic integration is unnecessary for function, sgRNA gene cassettes can be synthesized through the single-joint PCR method (11), thus minimizing investigators’ time and expense. In addition, we envision that multiple sgRNA expression cassettes might be transformed together to enable rapid genomic targeting of several loci at once, as demonstrated for integrated constructs by Vyas et al. (3). We note that transient expression of CRISPR components may minimize off-target cleavage activity and associated toxicity (12, 13).

We suggest that transiently transforming DNA expression may have additional uses. For example, one could screen pooled *C. albicans* mutants for altered green fluorescent protein (GFP) reporter gene expression by cell sorting after transformation of a nonintegrating GFP fusion gene. Also, one could assay for transient rescue of phenotypic defects in such properties as adherence, cell type switching, or mating with a suitable nonintegrating effector gene construct. Therefore, transient expression approaches may accelerate future functional analysis in *C. albicans*.

Our data indicate that CRISPR-targeted cleavage can occur far from sites of recombination, a result that is not surprising based on studies in yeast and mouse (7, 8). The finding is useful because it indicates that complete gene deletions are possible with the CRISPR system in *C. albicans*. Vyas et al. have defined unique sgRNA sequences computationally (3), with the goal of minimizing the potential for secondary-site cleavage, but not all of these ideal sgRNA sequences may lie in essential parts of coding regions. A complete gene deletion obviates the concern that a site mutation may cause only partial gene inactivation.

The fact that CRISPR-promoted homozygous mutations can occur through both independent integration events and single integration followed by allelic gene conversion has several implications for gene function analysis. Most importantly, the occurrence of allelic gene conversion may affect neighboring genes, and it will be prudent to validate homozygous mutant phenotypes. Such validation may be accomplished by complementation by the introduction of a wild-type copy of the gene of interest, as is currently the standard in the field. Complete complementation may not always be feasible, as in the case of multigene mutants. In such cases, the connection between genotype and phenotype is strengthened if several independent mutant isolates give consistent results. Fortunately, the CRISPR system facilitates the isolation of numerous homozygotes, so the construction of independent mutant isolates will be accelerated.

The transient CRISPR system for *C. albicans* (Fig. 2) may be used in the future as follows. The *CaCAS9* expression cassette may be PCR amplified from plasmid pV1093 (3). sgRNA expression cassettes may be designed for almost any gene with the sequences presented by Vyas et al. (3) and may be constructed through single-joint PCR (Fig. 3). Finally, mutant gene templates may be created through PCR amplification of a *NAT* gene, such as from plasmid pNAT, using flanking primers to direct insertion or deletion-insertion alleles (Fig. 2B). The use of auxotrophic *C. albicans* strains, such as BWP17 or SN152, will allow the use of nutritional markers, as well. Finally, transformation may be conducted with the unmarked *CaCAS9* and sgRNA gene PCR products and the deletion-insertion template PCR product, followed by selection for the relevant marker. We believe this transient CRISPR system will streamline gene function analysis in *C. albicans*.

## MATERIALS AND METHODS

### Strains and culture conditions.

*C. albicans* strains SC5314 (14), BWP17 (*ura3Δ*::λ*imm434*/*ura3*Δ::λ*imm434 his1*Δ::*hisG*/*his1*Δ::*hisG arg4*Δ::*hisG*/*arg4*Δ::*hisG*) (15), and a *yhb5* mutant derivative of strain SN152 referred to as Noble_ORF_NegativeMutantCollection_Mutant no. 643 (*his1*Δ/*his1*Δ *leu2*Δ/*leu2*Δ *arg4*Δ/*arg4*Δ *URA3*/*ura3*Δ::*imm434 IRO1*/*iro1*Δ::*imm434 yhb5Δ*::*CdHIS1*/*yhb5*Δ::*CmLEU2* [contains *HIS1* from *C. dubliniensis* and *LEU2* from *Candida maltosa*]) (10) were used as transformation recipients. Fungal strains were grown at 30°C in YPD+uri (2% Bacto peptone, 2% dextrose, 1% yeast extract, and 80 µg/ml uridine) with shaking. *C. albicans* transformants were selected on YPD+NAT (2% Bacto peptone, 2% dextrose, 1% yeast extract, 80 µg/ml uridine, and 400 µg/ml nourseothricin [NAT; Werner BioAgents]) for nourseothricin-resistant (Nat^R^) isolates or on synthetic medium (2% dextrose, 1.7% Difco yeast nitrogen base with ammonium sulfate and auxotrophic supplements). All strains were stored as glycerol stocks at −80°C.

### Plasmids/DNA.

To construct the pNAT plasmid, plasmid pCJN542 (16) was cut with SacI and SpeI to remove the *TDH3* promoter. The SacI-SpeI fragment containing the nourseothricin resistance cassette (*NAT*) was blunted and self-ligated (17) to yield plasmid pNAT. The plasmid pV1093 used in this study was a kind gift from Valmik Vyas (3). We cloned the 20-bp guide sequence for *ADE2* into the pV1093 vector, yielding pADE2-sgRNA. The *CaCAS9* gene was the *CAS9* gene that had been codon optimized for expression in *C. albicans* (3). The *CaCAS9* expression cassette containing the *ENO1* promoter, *CaCAS9* open reading frame (ORF), and *CYC1* terminator was PCR amplified from plasmid pV1093 (Fig. 2A). The sgRNA expression cassette containing the *SNR52* promoter, guide sequence, and sgRNA scaffold sequence was assembled by the single-joint PCR method (11). In the first step, the *SNR52* promoter and sgRNA scaffold components were PCR amplified using both flanking primers and internal chimeric primers (Fig. 3). The chimeric primers overlapped by a 20-base segment that specified the guide sequence. In the second step, both components were joined by primer extension, relying upon annealing of the complementary chimeric primer extensions. In the third step, the joined product was PCR amplified with nested primers to yield the sgRNA cassette (Fig. 3). Gene deletion PCR constructs were synthesized using plasmid pNAT or pRS-ARG4 (15) or the *CdARG4* plasmid pSN105 (10), modified slightly, as the template. The primers were designed to include 80 bases with homology to the sequences upstream or downstream from the target gene (Fig. 2B). The oligonucleotides used in this study are listed in Table S1 in the supplemental material. PCR was conducted with Ex *Taq* in accordance with the manufacturer’s instructions (TaKaRa Bio, Inc.).

10.1128/mSphere.00130-16.1 Table S1  Oligonucleotides used in this study. Download Table S1, DOCX file, 0.1 MB. Copyright © 2016 Min et al. 2016 Min et al. This content is distributed under the terms of the Creative Commons Attribution 4.0 International license.

### Fungal transformation.

PCR products for transformation were purified and concentrated with the GeneJET PCR purification kit (Thermo Fisher Scientific, Inc.). In the original *C. albicans* CRISPR system, the deletion constructs (8 µg) were cotransformed with the CRISPR expression plasmid (5 µg), which had been linearized by digestion with KpnI and SacI (3). In the transient CRISPR system, the deletion constructs (3 µg) were cotransformed with the *CaCAS9* cassette (1 µg) and sgRNA cassette (1 µg), using the lithium acetate transformation method (18). The transformation frequency was calculated as the ratio of the number of cells that form colonies on selective medium divided by the number on nonselective YPD medium.
