# Supplementary material for: Candida albicans Gene Deletion with a Transient CRISPR-Cas9 System
Source: mSphere. 2016 Jun 15;1(3):e00130-16. doi: 10.1128/mSphere.00130-16 (PMC4911798; doi:10.1128/mSphere.00130-16)
Supplement: Table S1 [file sph003162103st1.docx]

**Supplementary Table 1.** Oligonucleotides used in this study

| **Primer** | **Sequence** | **Description** |
| --- | --- | --- |
| CaCas9/for | ATCTCATTAGATTTGGAACTTGTGGGTT | Forward and reverse primers for amplification of *CaCas9* cassette |
| CaCas9/rev | TTCGAGCGTCCCAAAACCTTCT |  |
| SNR52/F | AAGAAAGAAAGAAAACCAGGAGTGAA | Forward primer for amplification of *SNR52* promoter |
| SNR52/R_ADE2 | attaggtcgtatgattgttgCAAATTAAAAATAGTTTACGCAAGTC | Reverse primer for amplification of *SNR52* promoter with overlapping ADE2.1 guide sequence |
| sgRNA/F_ADE2 | caacaatcatacgacctaatGTTTTAGAGCTAGAAATAGCAAGTTAAA | Forward primer for amplification of sgRNA scaffold with overlapping ADE2.1 guide sequence |
| sgRNA/R | ACAAATATTTAAACTCGGGACCTGG | Reverse primer for amplification of sgRNA scaffold |
| SNR52/N | GCGGCCGCAAGTGATTAGACT | Forward and reverse nested primers for third round PCR for construction of sgRNA expression cassette |
| sgRNA/N | GCAGCTCAGTGATTAAGAGTAAAGATGG |  |
| SNR52/R_ADE2_2 | aaggggttcactgaattctcCAAATTAAAAATAGTTTACGCAAGTC | Reverse primer for amplification of *SNR52* promoter with overlapping ADE2.2 guide sequence |
| sgRNA/F_ADE2_2 | gagaattcagtgaaccccttGTTTTAGAGCTAGAAATAGCAAGTTAAA | Forward primer for amplification of sgRNA scaffold with overlapping ADE2.2 guide sequence |
| ADE2_del_2F | ttattctcatcacacacgcatatacaagcactacacataatggatagcaaaactgttggtattttaggaggtTAATGAttTTTCCCAGTCACGACGTT | Forward primer for amplification of *ADE2* deletion cassette |
| ADE2_del_2R | ccgaaagtattatcaactaagaagggaaaagcaccacattttctaatgtctcatatattagttcagttcgatgtatttctGTGGAATTGTGAGCGGATA | Reverse primer for amplification of *ADE2* deletion cassette |
| ADE2-fwd | aacaccccccaccaaaaagaatc | Forward primer at -89 bp site of *ADE2* ORF |
| ADE2-rev | acaagtcatcgactgtgttgg | Reverse primer at +403 bp site of *ADE2* ORF |
| NAT/rev | TCAATGGTGGATCAACTGGAACTTC | Reverse primer to detect *NAT* expression cassette |
| sgRNA_check/F | GGCTCGAACACAGTACCTCCAGA | Forward primer at -886 bp site of guide sequence to detect sgRNA expression cassette |
| sgRNA_check/R | GGCGGCAAAACTAATTCTTCTCTT | Reverse primer at +166 bp site of guide sequence to detect sgRNA expression cassette |
| pV1093_seq_3F | AATTATCAAAAGACACCTATGACGACG | Forward primer at +793 bp site of CaCas9 ORF to detect *CaCas9* expression cassette |
| pV1093_seq_4R | TCAACTGTTTCATCACTTTATCGTCAA | Reverse primer at +1954 bp site of CaCas9 ORF to detect *CaCas9* expression cassette |
| SNR52/R_ FRP1 | tttgatcaacagttttttgtCAAATTAAAAATAGTTTACGCAAGTC | Reverse primer for amplification of *SNR52* promoter with overlapping FRP1 guide sequence |
| sgRNA/F_FRP1 | acaaaaaactgttgatcaaaGTTTTAGAGCTAGAAATAGCAAGTTAAA | Forward primer for amplification of sgRNA scaffold with overlapping FRP1 guide sequence |
| FRP1_CRISPR-for | aatgaaaagtattaaacaaatctatattcacctctaaatttttttttttttttatcattaaccaagtttaactttcaaccACATTTCTGTACCGCAAATGTATCG | Forward primer for amplification of *FRP1* deletion cassette |
| FRP1_CRISPR-rev | atgggggtgtgtccttacgtacaggaatttatgtccgccttgctaaaacatacacaaaccttaaatttatcctatataccAGTAATGAAAGCAGTCAAAGGGCTC | Reverse primer for amplification of *FRP1* deletion cassette |
| FRP1-fwd | CATCCCTTGTCTGATGATAATAAGG | Forward primer at -120 bp site of *FRP1* ORF |
| FRP1-rev | TTTGTTATTGACTCGCAAGTATCGTG | Reverse primer at +150 bp site of *FRP1* ORF |
| ARG4-rev | TTATCTTAACCTTCTAGTCCACGT | Reverse primer to detect *CdARG4* expression cassette |
